# Clinical outcomes of percutaneous transhepatic biliary drainage at different Couinaud's hepatic entry segments for treating obstructive jaundice

**DOI:** 10.3389/fsurg.2023.1039106

**Published:** 2023-01-24

**Authors:** Ya-Chun Hsu, Hsing-Yu Lee, Chia-Ming Chang, Chia-Ying Lin, Yi-Sheng Liu, Han-Sheng Huang

**Affiliations:** Department of Medical Imaging, National Cheng Kung University Hospital, Tainan, Taiwan

**Keywords:** couinaud, hepatic segment, hepatic lobe, obstructive jaundice, complications

## Abstract

**Introduction:**

Percutaneous transhepatic biliary drainage (PTBD) is a common procedure for biliary obstructive jaundice caused by biliary tract obstruction. In clinical practice, PTBD can be carried out at right- or left-sided approach. However, different hepatic entry site may affect success rates and complications. Couinaud classification of liver anatomy further divides the liver into functionally independent segments (segment 2/3, segment 5/6, and segment 7/8). Therefore, this study aimed to elucidate whether different Couinaud hepatic segments as PTBD entry site are associated with high PTBD success and low complications.

**Methods:**

A total of 617 patients who underwent PTBD were retrospectively reviewed. Univariate and multivariate logistic regression analyses were performed to identify entry segments associated with PTBD success, bilirubin reduction, and complications.

**Results:**

With higher hepatic segment of PTBD entry site (segment 2/3, 5/6, and 7/8), the trend of PTBD success rate (82.0%, 71.7% and 60.7%; P<0.001) and bilirubin reduction (93.2%, 89.5%, and 82.0%; P=0.012) decreased. Furthermore, PTBD entry at segment 7/8 (42.6%) had highest complication rate than segment 5/6 (6.4%) and 2/3 (9.4%). Univariate and multivariate logistic regression analyses showed that PTBD entry segment was an independent factor associated with PTBD success, bilirubin reduction, and complications. Compared to segment 7/8, segment 2/3 and 5/6 had higher odds of PTBD success (aOR=2.699 and aOR=1.454, respectively) and bilirubin reduction (aOR=3.472 and aOR=2.361, respectively) and associated with lower risk of complications (aOR=0.143 and aOR=0.098, respectively). No independent risk factor for PTBD success and bilirubin reduction were identified in intrahepatic tumors. Moreover, for extrahepatic tumors, PTBD entry at segment 2/3 and segment 5/6 was more likely achieve PTBD success (aOR=3.037 and aOR=1.929, respectively), bilirubin reduction (aOR=3.069 and aOR=3.515) and low complications (aOR=0.102 and aOR=0.126, respectively).

**Discussion:**

Good clinical outcomes were observed for PTBD entry at segments 5/6 and 2/3. In contrast, segment 7/8 had the lowest success rate, smallest bilirubin reduction, and the highest complication rate. For patients with obstructive jaundice, PTBD entry in hepatic segments 2/3 and 5/6 is recommended to achieve high success rates and low complications.

## Introduction

Obstructive jaundice occurs when blockage of the biliary drainage system prevents the passage of bile from the liver to the small intestine, leading to conjugated hyperbilirubinemia. The most common causes of biliary tree obstruction are malignancies, gallstones, and fibrosis, manifesting symptoms as pain, nausea, vomiting, and jaundice (1). Such obstructions affect a large portion of the population worldwide, with impaired quality of life and significant morbidity and mortality (1–3). In the US, the incidence of biliary obstruction is approximately 5 cases per 1,000 people (4). The symptoms of obstructive jaundice, including abdominal pain, cholangitis, and pruritus, can be relieved by procedures that decompress the affected areas of the biliary circulation (5). Although surgical resection of malignant obstructive jaundice or endoscopic removal of gallstones is usually the mainstay of treatment, it is not always feasible due to the small number of eligible patients and the higher incidence of postoperative complications.

With advances in technology, percutaneous transhepatic biliary drainage (PTBD) has been considered as an effective palliative treatment of obstructive jaundice, especially when surgery is assessed to be infeasible (6, 7). PTBD is used to decompress intra- and extrahepatic biliary ducts in cases of malignant or benign obstruction to alleviate symptoms, decrease serum bilirubin before starting chemotherapy, or to facilitate biliary stent placement (5). Under imaging guidance using fluoroscopy with or without ultrasound, a needle is used to puncture the liver at the chosen location and is directed to the appropriate segmental duct (5). Nevertheless, the in-hospital mortality rate in patients receiving PTBD for obstructive jaundice is still high, with even reported mortality rates as high as nearly 20% (8, 9). For unresectable biliary tract obstruction, higher mortality is associated with preoperative clinical factors (eg, older patients, men, and these with increasing comorbidities) and, in particular, with inexperienced operator (lower-volume PTBD providers) (9).

PTBD can be performed *via* either the right (subcostal or intercostal) or left ductal (subxiphoid) approach. The chosen approach is presently viewed as a personal preference, with advantages and disadvantages for each (5). However, the few studies comparing PTBD efficacy, effects on quality of life, and complications between the two approaches report conflicting results. A randomized controlled trial including 64 patients found that self-reported quality of life following PTBD was higher for left than for right access (10). In contrast, a study including 50 PTBD patients reports no significant difference in quality of life, success rates, or complications between the left and right approaches (11). Other studies investigating complications after PTBD report a slightly higher rate of hemobilia following the left approach (12) and more cases of cholangitis and fever with the right approach (13), but the differences were not statistically significant in either study. While one study of 36 PTBD procedures observed no differences in efficacy or safety between the 2 approaches(14), our previous study including 446 PTBD patients showed that the left approach had better clinical outcomes and fewer complications (15).

Several factors may contribute to these conflicting results. Many of the study cohorts were relatively small, lowering the statistical power. In addition, the anatomical classification of the liver entry site as left or right lobe may be too broad to identify entry-site effects. The Couinaud system of liver classification divides the liver into 8 functionally independent segments, each with its own vascular inflow, biliary drainage, and lymphatic drainage (16, 17). Segments 2–4 compose the left hepatic lobe, and segments 5–8 compose the right hepatic lobe. No study has investigated the effect of PTBD entry site using liver segments to designate the entry site. Therefore, this study aims to expand the understanding of the treatment outcomes of patients with obstructive jaundice according to different segments of PTBD entry. In a large cohort of 617 patients, we determine and compare the technical success rates, degree of bilirubin reduction, and complication rates between PTBD entry in segments 2/3, 5/6, and 7/8.

## Materials and methods

### Study design and patients

This retrospective study was approved by the Institutional Review Board (IRB) of National Cheng Kung University Hospital (IRB number: B-ER-110-392). The requirement of written informed consent was waived by the IRB due to the retrospective nature of the study. Patients who presented to National Cheng Kung University Hospital with obstructive jaundice between 2008 and 2020 were retrospectively screened. Inclusion criteria were age > 20 years and treatment with PTBD. Exclusion criteria were missing clinical information (e.g., demographic data, procedure time, duct diameter, indications for obstruction, presence of ascites, obstruction level, hepatic puncture site) and missing data regarding final treatment outcomes (PTBD success and bilirubin reduction) or complications. In this study, all PTBD procedures were conducted with placement of an around 8 Fr small pigtail drainage catheter and were performed by interventional radiologists with more than 10 years of experience in biliary intervention. Postoperative complications were graded according to the Clavien-Dindo classification. Bilirubin reduction was defined as normalization of bilirubin or a serum total bilirubin concentration of ≤3 mg/dl or decrease in bilirubin levels by ≥30% within 2 weeks of biliary drainage.

### Statistical analysis

All statistical analyses were performed using IBM SPSS statistical software version 22 for Windows (IBM Corp., Armonk, New York, USA). Continuous variables are expressed as the mean ± standard deviation (SD), and categorical variables are presented as frequency and percentage. Differences between continuous variables were examined using one-way ANOVA. Categorical variables were compared using the Chi-square test or Fisher's exact test. Univariate and multivariate logistic regression analyses were performed to identify risk factors associated with PTBD success, bilirubin reduction, and complications. Results are expressed as the odds ratio (OR) with 95% confidence interval (CI). Stratified analyses were performed based on malignancy and adjusted for significant risk factors identified in univariate analysis. Two-sided *P* < 0.05 was considered statistically significant.

## Results

### Patient characteristics and PTBD outcomes and complications

Demographic data and clinical outcomes of 617 patients with obstructive jaundice who received PTBD are shown in Table 1. The mean patient age was 67.14 ± 13.61 years, and 55.6% of the patients were male. Most patients (85.6%) underwent PTBD in the hospital ward. The mean targeted bile duct diameter was 5.30 ± 2.55 cm, and the mean PTBD procedure time was 43.92 ± 78.70 min. The most common indication for PTBD was malignant biliary obstruction (79.1%), and the remainder were stones (11.8%) and fibrosis (9.1%). Most of the 488 malignant biliary obstructions were located in the extrahepatic (369; 77.0%) or intrahepatic (112; 23.0%) bile ducts. Obstructions were at the common bile duct (CBD) (45.2%), common hepatic duct (CHD) (32.3%), lobar (20.7%), and segmental (1.8%) levels. Only a subset of patients had a small (7%) or moderate/massive amount (3.6%) of ascites before PTBD. The PTBD puncture was performed at the segmental (31.2%), subsegmental (51.8%), or sub-subsegmental (17.0%) level of the intrahepatic duct. Based on the Couinaud classification of liver segments, the patients were further grouped according to the liver segment of PTBD entry, as follows: segment 2/3, *N* = 383; segment 5/6, *N* = 173; and segment 7/8, *N* = 61. No statistically significant difference was observed in age, sex, surgery time, surgical department, obstruction level, or ascites between the three groups (all *P* > 0.05). In addition, no significant difference in malignant extrahepatic or extrahepatic bile duct obstruction was observed between the three groups (*P* = 0.061). The PTBD liver puncture levels differed significantly between the three groups (*P* < 0.001). The puncture level in the segment 2/3 group was located primarily in the intrahepatic subsegmental ducts (60.8%), followed by segmental (25.8%) and sub-subsegmental ducts (13.3%). In contrast, the most common puncture level in the Segment 7/8 group was segmental ducts (65.6%), followed by subsegmental (29.5%) and sub-subsegmental ducts (4.9%). In the Segment 5/6 group, the proportion of puncture levels in segmental, subsegmental and sub-subsegmental ducts was relatively equal.

**Table 1:** Patient characteristics and surgical outcomes of PTBD.

	Total	Segment 2, 3	Segment 5, 6	Segment 7, 8	*P*
	*N* = 617	*N* = 383	*N* = 173	*N* = 61	
Age	67.14 ± 13.61	67.07 ± 13.82	66.43 ± 12.93	69.57 ± 14.13	0.298
Sex					0.529
Female	274 (44.4%)	176 (46.0%)	73 (42.8%)	24 (39.3%)	
Male	343 (55.6%)	207 (54.0%)	99 (57.2%)	37 (60.7%)	
Procedure time	43.92 ± 78.70	44.43 ± 86.08	44.78 ± 74.76	37.90 ± 15.75	0.822
Duct diameter	5.30 ± 2.55	5.69 ± 2.73	4.37 ± 2.03	5.53 ± 2.01	**<0.001**
Department					0.781
Ward	528 (85.6%)	326 (85.1%)	147 (85.5%)	54 (88.5%)	
ER	89 (14.4%)	57 (14.9%)	25 (14.5%)	7 (11.5%)	
Indication					**0.001**
Stone	73 (11.8%)	37 (9.7%)	19 (11.0%)	17 (27.9%)	
Fibrosis	56 (9.1%)	32 (8.4%)	18 (10.4%)	6 (9.8%)	
Malignancy	488 (79.1%)	314 (82%)	135 (78.6%)	38 (62.3%)	0.061
Intrahepatic	112 (23.0%)	63 (20.1%)	39 (28.7%)	10 (26.3%)	
Extrahepatic	376 (77.0%)	251 (79.9%)	96 (71.3%)	28 (73.7%)	
Obstruction level					0.174
CBD	279 (45.2%)	171 (44.6%)	76 (43.9%)	32 (52.5%)	
CHD	199 (32.3%)	135 (35.2%)	47 (27.2%)	17 (27.9%)	
Lobar	128 (20.7%)	70 (18.3%)	46 (26.6%)	12 (19.7%)	
Segmental	11 (1.8%)	7 (1.8%)	4 (2.3%)	0 (0.0%)	
Ascites					0.856
No	552 (89.5%)	340 (88.8%)	155 (89.6%)	57 (93.4%)	
Small amount	43 (7.0%)	28 (7.3%)	12 (6.9%)	3 (4.9%)	
Moderate/massive amount	22 (3.6%)	15 (3.9%)	6 (3.5%)	1 (1.6%)	
Entry level					**<0.001**
Segmental	192 (31.2%)	99 (25.8%)	53 (30.6%)	40 (65.6%)	
Subsegmental	319 (51.8%)	233 (60.8%)	68 (39.3%)	18 (29.5%)	
Sub-subsegmental	105 (17.0%)	51 (13.3%)	51 (29.5%)	3 (4.9%)	

The clinical outcomes, including PTBD success, bilirubin reduction, and complications, are shown in Table 2. The PTBD success rate tended to decrease with increasing PTBD hepatic segment height (segment 2/3, 82.0%; segment 5/6, 71.7%; segment 7/8, 60.7%; *P* < 0.001). The observed PTBD success rates were consistent with the level of serum bilirubin reduction (segment 2/3, 93.2%; segment 5/6, 89.5%; segment 7/8, 82.0%; *P* = 0.012). However, the segment 7/8 group had the highest complication rate of 42.6%, while the segment 5/6 group had the lowest complication rate of only 6.4%. The most frequent PTBD complications in the total cohort were post-procedure blood clot (31.5%), fever/infection (21.9%), and gross hemobilia (19.2%). Most complications were grade I-II. Only 10 patients experienced complications of Clavien-Dindo grade III. No patients underwent PTBD had severe complications (grade > IV) based on the Clavien–Dindo classification. In the Segment 2/3 group, blood clot (36.1%) was the most frequent complication. Fever/infection (45.5%) was the most frequent complication in the segment 5/6 group, while pleural effusion (38.5%) was the most frequent complication in the segment 7/8 group. In summary, PTBD entry in hepatic segment 5/6 had similar good success rates and low complication rates as did segment 2/3 entry. PTBD entry in hepatic segment 7/8 was associated with the lowest success rates and highest complication rates.

**Table 2:** Clinical outcomes of PTBD success, bilirubin reduction, and complications.

	Total	Segment 2, 3	Segment 5, 6	Segment 7, 8	*P*
	*N* = 617	*N* = 383	*N* = 172	*N* = 61	
PTBD success					**<0.001**
No	142 (23.0%)	69 (18.0%)	49 (28.3%)	24 (39.3%)	
Yes	475 (77.0%)	314 (82.0%)	124 (71.7%)	37 (60.7%)	
Bilirubin reduction					**0.012**
No	55 (8.9%)	26 (6.8%)	18 (10.5%)	11 (18.0%)	
Yes	561 (91.1%)	357 (93.2%)	154 (89.5%)	50 (82.0%)	
Complications					**<0.001**
No	544 (88.2%)	347 (90.6%)	162 (93.6%)	35 (57.4%)	
Yes	73 (11.8%)	36 (9.4%)	11 (6.4%)	26 (42.6%)	
Post-procedure blood clot	23 (31.5%)	13 (36.1%)	3 (27.3%)	7 (26.9%)	
Fever/infection	16 (21.9%)	8 (22.2%)	5 (45.5%)	3 (11.5%)	
Bleeding *via* PTBD	3 (4.1%)	1 (2.8%)	0 (0.0%)	2 (7.7%)	
Gross hemobilia	14 (19.2%)	8 (22.2%)	1 (9.1%)	5 (19.2%)	
Pleural effusion	11 (15.1%)	1 (2.8%)	0 (0.0%)	10 (38.5%)	
Bile leaks	10 (13.7%)	5 (13.9%)	3 (27.3%)	2 (7.7%)	
Abdominal bleeding	2 (2.7%)	1 (2.8%)	1 (9.1%)	0 (0.0%)	
Others	1 (1.4%)	1 (2.8%)	0 (0.0%)	0 (0.0%)	
Removal reason					0.193
Maturity	34 (5.5%)	24 (6.3%)	5 (2.9%)	5 (8.2%)	
Dislodgement	86 (13.9%)	48 (12.5%)	23 (13.4%)	15 (24.6%)	
Obstruction	99 (16.0%)	57 (14.9%)	34 (19.8%)	8 (13.1%)	
Leakage	12 (1.9%)	6 (1.6%)	4 (2.3%)	2 (3.3%)	
Hemorrhage	7 (1.1%)	7 (1.8%)	0 (0.0%)	0 (0.0%)	
Death	118 (19.1%)	81 (21.1%)	30 (17.4%)	7 (11.5%)	
Unnecessary/other operation	217 (35.2%)	130 (33.9%)	66 (38.4%)	20 (32.8%)	
Infection	9 (1.5%)	6 (1.6%)	2 (1.2%)	1 (1.6%)	
Other	34 (5.5%)	23 (6.0%)	8 (4.7%)	3 (4.9%)	

### Factors associated with PTBD success, bilirubin decrease, and complications

Univariate and multivariate logistic regression analyses were performed to identify independent risk factors for PTBD success, bilirubin reduction, and complications. As shown in Table 3, ascites was an independent risk factor for bilirubin reduction. Absence of ascites before PTBD was significantly associated with a higher rate of bilirubin reduction (aOR, 3.437; 95% CI, 1.703–6.937; *P*, 0.001). In addition, the liver segment of PTBD entry was an independent risk factor associated with PTBD success, bilirubin reduction, and complications. Compared to Segment 7/8, the adjusted odds of PTBD success were significantly higher in with Segment 2/3 entry (aOR, 2.699; 95% CI, 1.484–4.908; *P*, 0.001). Segment 5/6 entry also had a higher odds ratio, although it did not reach statistical significance (aOR, 1.454; 95% CI, 0.884–3.000; *P*, 0.246). Similarly, entry at Segment 2/3 (aOR, 3.472; 95% CI, 1.584–7.609; *P*, 0.002) and Segment 5/6 (aOR, 2.361; 95% CI, 1.013–5.503; *P*, 0.047) had significantly higher adjusted odds of bilirubin reduction than did Segment 7/8. The odds of complications were higher for Segment 7/8 entry than for Segment 2/3 (aOR, 0.143; 95% CI, 0.074–0.276; *P* < 0.001) and Segment 5/6 (aOR, 0.098; 95% CI, 0.043–0.224; *P* < 0.001). These results suggest that hepatic segments 2/3 and 5/6 are better PTBD entry sites for optimizing success rates and minimizing complications.

**Table 3:** Logistic regression analysis of factors associated with PTBD success, bilirubin reduction, and complications.

	Bilirubin decreased				PTBD success				Complications			
	OR	*P*	aOR	*P*	OR	*P*	aOR	*P*	OR	*P*	aOR	*P*
Age (years)	0.989	0.303			0.988	0.107			1.007	0.436		
Sex												
Male	Ref.				Ref.				Ref.		Ref.	
Female	1.320	0.338			1.115	0.572			0.578	**0**.**038**	0.582	0.051
Duct diameter (mm)	1.125	0.079	1.132	0.079	1.013	0.735			0.949	0.321		
Indication												
Malignancy	Ref.				Ref.				Ref.			
Stone	1.843	0.255			1.492	0.216			1.02	0.958		
Fibrosis	1.389	0.544			1.487	0.276			0.711	0.486		
Obstruction level												
CBD	Ref.				Ref.				Ref.			
CHD	1.249	0.526			0.999	0.995			0.878	0.649		
Lobar	0.661	0.227			0.677	0.109			0.760	0.423		
Segmental	0.945	0.958			1.239	0.788			0.672	0.709		
Ascites												
Yes	Ref.		Ref.		Ref.				Ref.			
No	3.030	**0**.**002**	3.437	**0**.**001**	1.563	0.121			1.361	0.491		
Entry segment												
Segment 7, 8	Ref.		Ref.		Ref.		Ref.		Ref.		Ref.	
Segment 5, 6	1.882	0.128	2.361	**0**.**047**	1.628	0.118	1.454	0.246	0.092	**<0**.**001**	0.098	**<0**.**001**
Segment 2, 3	3.021	**0**.**005**	3.472	**0**.**002**	2.952	**<0**.**001**	2.699	**0**.**001**	0.140	**<0**.**001**	0.143	**<0**.**001**
Entry level												
Segmental	Ref.				Ref.		Ref.		Ref.			
Subsegmental	0.714	0.300			1.472	0.067	1.209	0.392	0.687	0.158	1.046	0.881
Sub-subsegmental	1.406	0.495			1.565	0.125	1.484	0.195	0.386	**0**.**030**	0.700	0.448

Biliary tract malignancies are primarily tumors of the intrahepatic and extrahepatic bile ducts. Promoting biliary drainage to decompress the biliary tract and improve quality of life is one goal of biliary malignancy treatment. Accordingly, we compared the factors associated with PTBD success, bilirubin reduction, and complications between patients with intra- and extrahepatic tumors. Multivariate logistic regression analysis showed that PTBD entry in segment 2/3 was more likely to achieve PTBD success (aOR, 3.037; 95% CI, 1.306–7.058; *P*, 0.010) and bilirubin reduction (aOR, 3.069; 95% CI, 1.011–9.314; *P*, 0.048) in patients with extrahepatic tumors (Table 4). For malignancies in the extrahepatic bile duct, PTBD entry in segment 5/6 also had a higher likelihood of bilirubin reduction (aOR, 3.515; 95% CI, 0.974–12.684; *P*, 0.055). No independent risk factors for bilirubin reduction or PTBD success were identified in malignancies in the intrahepatic bile duct. PTBD entry in segments 2/3 and 5/6 had a lower likelihood of complications in both intrahepatic tumors (aOR, 0.142; 95% CI, 0.027–0.742; *P*, 0.021 and aOR, 0.114; 95% CI, 0.015–0.848; *P*, 0.034, respectively) and extrahepatic tumors (aOR, 0.126; 95% CI, 0.051–0.310; *P* < 0.001 and aOR, 0.102; 95% CI, 0.035–0.298; *P* < 0.001, respectively). In addition, ascites before PTBD remained an independent risk factor for bilirubin reduction in patients with extrahepatic tumors, with an adjusted odds ratio of 3.363 (95% CI, 1.356–8.342; *P*, 0.009).

**Table 4:** Adjusted association of intrahepatic/extrahepatic tumors with PTBD success, bilirubin reduction, and complications.

	Intrahepatic tumor		Extrahepatic tumor	
	aOR	*P*	aOR	*P*
**PTBD success**				
Entry segment (ref. Segment 7, 8)				
Segment 5, 6	0.454	0.314	1.929	0.154
Segment 2, 3	1.711	0.484	3.037	**0**.**01**
Entry level (ref. Segmental)				
Subsegmental	1.132	0.806	0.977	0.934
Sub-subsegmental	1.476	0.529	1.744	0.183
**Bilirubin reduction**				
Duct diameter (mm)	0.988	0.922	1.193	0.086
Ascites (No vs. Yes)	1.19	0.812	3.363	**0**.**009**
Entry segment (ref. Segment 7, 8)				
Segment 5, 6	1.676	0.523	3.515	**0**.**055**
Segment 2, 3	5.013	0.053	3.069	**0**.**048**
**Complications**				
Sex (female vs. male)	0.606	0.466	0.556	0.098
Entry segment (ref. Segment 7, 8)				
Segment 5, 6	0.114	**0**.**034**	0.102	**<0**.**001**
Segment 2, 3	0.142	**0**.**021**	0.126	**<0**.**001**
Entry level (ref. Segmental)				
Subsegmental	3.801	0.124	0.803	0.563
Sub-subsegmental	–	0.998	0.766	0.615

## Discussion

In a large cohort of patients who underwent PTBD for obstructive jaundice, we observed that the liver segment of PTBD entry is an independent factor associated with PTBD success, bilirubin reduction, and complications. Equally good clinical outcomes (high PTBD success and bilirubin reduction; low complication rates) were observed for entry at liver segment 5/6 and liver segment 2/3. However, PTBD entry at liver segment 7/8 was associated with the lowest success rate, smallest bilirubin reduction, and the highest rate of complications. These results suggest that PTBD entry in hepatic segments 2/3 and 5/6 optimizes success rates and minimizes complications. As the first study to investigate PTBD success based on Couinaud liver segment classification, these results confirm that such entry site designation provides more detailed information regarding PTBD success according to entry site than does left/ right lobe designation.

The clinical success rate of PTBD (77.0%) for our total cohort is comparable to that of other studies, reporting rates of 87.5% (18), 76.5% (19), and 76% (11). Importantly, we observed that the clinical success of PTBD varied with the site of entry. While our previous study showed a significantly greater PTBD clinical success rate for left than right lobe access (15), the segmental designation used in the present study allowed us to identify a significant difference in success rates for sites within the right lobe. Entry into liver segment 7/8 (right lobe) had a significantly lower clinical success rate (60.7%) than did segments 5/6 (right lobe) (71.7%) and 2/3 (left lobe) (82%). This finding indicates further access options for successful PTBD, including segments 2/3 as well as 5/6.

We observed significantly lower complication rates with PTBD entry at segment 2/3 (9.4%) and 5/6 (6.4%) than at segment 7/8 (42.6%). Rafiq et al. observed more complications with right-sided (subcostal or intercostal) than left-sided (sub-xiphoid) PTBD, although the differences were statistically insignificant, possibly due to their small cohort size (*n* = 31) (13). The similar result in our much larger cohort not only confirms that fewer complications occur in left-side PTBD but also shows that the high complication rate for right-sided PTBD is due to entry at segment 7/8. One possible reason for the high complication rate associated with segment 7/8 entry is that this region is affected by respiratory movements. Catheters may become malpositioned in response to respiratory movements and can gradually be pulled out of the liver. One study reports that PTBD catheter dislodgement occurred only on the right side, noting that its displacement was due to continuous motion of the catheter in the intercostal space during respiration (13). Another study also reports a higher incidence of catheter dislodgement in right-sided PTBD, and the authors suggest that the high incidence may be due to the small volume of the intercostal space and greater movement of the catheter within the liver during respiration, leading to displacement of the internal section of the catheter (11). PTBD entry at Segment 5/6 is thus recommended in cases involving an atrophic or high-riding left hepatic lobe (segment 2/3).

This study has several limitations. The retrospective nature of this study limits the data available for analysis, including information regarding quality of life after PTBD. The PTBD procedures were performed by many operators with varied levels of experience, which may have affected the PTBD success rates or complication rates.

## Conclusion

PTBD entry in hepatic segment 7/8 has the lowest clinical success rate and highest complication rate. Despite previous reports of lower PTBD efficacy for right-sided entry, we observed that PTBD entry in hepatic segments 2/3 (left hepatic lobe) or 5/6 (right hepatic lobe) optimizes clinical success and minimizes complications. For studies of PTBD efficacy, Couinaud liver segment classification of entry sites provides more detailed results than does left/ right lobe designation.

## Data Availability

The original contributions presented in the study are included in the article/Supplementary Material, further inquiries can be directed to the corresponding author/s.
